# Radiogenomic Predictors of Recurrence in Glioblastoma—A Systematic Review

**DOI:** 10.3390/jpm12030402

**Published:** 2022-03-04

**Authors:** Felix Corr, Dustin Grimm, Benjamin Saß, Mirza Pojskić, Jörg W. Bartsch, Barbara Carl, Christopher Nimsky, Miriam H. A. Bopp

**Affiliations:** 1 Department of Neurosurgery, University of Marburg, Baldingerstrasse, 35043 Marburg, Germany; dustin.grimm@students.edu.edu.mt (D.G.); sassb@med.uni-marburg.de (B.S.); mirza.pojskic@uk-gm.de (M.P.); jbartsch@med.uni-marburg.de (J.W.B.); barbara.carl@helios-gesundheit.de (B.C.); nimsky@med.uni-marburg.de (C.N.); bauermi@med.uni-marburg.de (M.H.A.B.); 2 EDU Institute of Higher Education, Villa Bighi, Chaplain’s House, KKR 1320 Kalkara, Malta; 3 Center for Mind, Brain and Behavior (CMBB), 35043 Marburg, Germany; 4 Department of Neurosurgery, Helios Dr. Horst Schmidt Kliniken, Ludwig-Erhard-Strasse 100, 65199 Wiesbaden, Germany

**Keywords:** radiogenomics, glioblastoma, GBM, recurrence, imaging genomics, radiomics, magnetic resonance imaging, gliomas, molecular markers, radiology, neuro-oncology

## Abstract

Glioblastoma, as the most aggressive brain tumor, is associated with a poor prognosis and outcome. To optimize prognosis and clinical therapy decisions, there is an urgent need to stratify patients with increased risk for recurrent tumors and low therapeutic success to optimize individual treatment. Radiogenomics establishes a link between radiological and pathological information. This review provides a state-of-the-art picture illustrating the latest developments in the use of radiogenomic markers regarding prognosis and their potential for monitoring recurrence. Databases PubMed, Google Scholar, and Cochrane Library were searched. Inclusion criteria were defined as diagnosis of glioblastoma with histopathological and radiological follow-up. Out of 321 reviewed articles, 43 articles met these inclusion criteria. Included studies were analyzed for the frequency of radiological and molecular tumor markers whereby radiogenomic associations were analyzed. Six main associations were described: radiogenomic prognosis, MGMT status, IDH, EGFR status, molecular subgroups, and tumor location. Prospective studies analyzing prognostic features of glioblastoma together with radiological features are lacking. By reviewing the progress in the development of radiogenomic markers, we provide insights into the potential efficacy of such an approach for clinical routine use eventually enabling early identification of glioblastoma recurrence and therefore supporting a further personalized monitoring and treatment strategy.

## 1. Introduction

Despite well-established multimodal therapeutic regimens, glioblastoma (GBM) is the most lethal primary brain tumor with low survival rates [1] that have not significantly improved over the last few years. Notably, more than half of patients will undergo tumor progression after six months following surgery [2,3], and the median survival is between ten and twelve months [4,5]. Magnetic resonance imaging (MRI) plays an essential role in diagnosing and monitoring tumor progression and therapy. Thus, in addition to preoperative diagnostic information, the extent of resection can be assessed early after surgery. Even though comprehensive imaging modalities for MRI are available, there is still no standard method that can reliably detect early tumor progression. A commonly used marker for tumor progression in higher grade gliomas is contrast enhancement [6]. However, parameters such as flow rates from perfusion measurements [7] also appear to be related to tumor progression and angiogenesis. In addition, there are parameters from MR spectroscopy to distinguish recurrence/progression and necrosis during therapy [8].

When assessing prognosis and selecting an individually appropriate treatment strategy, prognostic and predictive molecular markers play an increasingly significant role [9] and have prompted significant changes to the 2016 World Health Organization (WHO) classification of central nervous system (CNS) tumors and refinements over the last years [10,11]. According to the new WHO classification published in 2021, only isocitrate dehydrogenase wildtype and H3 wildtype (IDH_wt_H3_wt_) astrocytomas are now classified as the GBM WHO grade 4 [11].

GBMs with similar imaging characteristics may exhibit different clinical courses, treatment responses, and outcomes [12] whilst genetically similar GBM tumors may present varying radiological characteristics morphologically distinct on MRI [13,14]. Additionally, investigations on the molecular level can provide little information regarding macroscopic characteristics, such as vascularization or space-occupying effects. In an era of precision medicine, intensive research has been conducted on imaging markers derived from routine clinical MR images to match with observations regarding molecular features of GBM tumors in a non-invasive manner. By taking a personalized view of the disease, precision medicine approaches aim to enable individualized decision making about the diagnostic and therapeutic approaches by using multiple data sources—from genomics to radiological sequences [15,16]. However, despite those numerous efforts in recent years, there still seems to have been no significant breakthrough in determining interrelated molecular and radiological characteristics that can be accurately used for individual prognostic stratification in GBM patients.

### 1.1. Prognosis of Disease Progression in Glioblastoma

Multiple factors have been shown to be relevant for the prognosis of disease progression. At the individual patient level, the pre-operative Karnofsky Performance Score (KPS) plays an important role, along with age and sex [17,18,19,20,21,22]. From a surgical point of view, location and accessibility are essential for maximally safe resection. Clinical studies have shown that the extent of maximum safe resection in glioblastoma therapy correlates with the progression-free interval (PFS) and overall survival (OS) [23,24,25]. To improve the extent of resection, the role of neuronavigation and intraoperative MRI could be demonstrated [26].

The prognosis in GBM is highly influenced by tumor biological characteristics. O6-methylguanine-DNA methyl-transferase (MGMT) promoter methylation is considered the most important predictive tool in adjuvant treatment with alkylating chemotherapeutic agents such as temozolomide, thus influencing treatment response and prognosis [12]. A methylated MGMT promoter correlates with increased survival time and recurrence pattern [27,28,29]. IDH mutations possess the greatest prognostic significance in gliomas and are associated with longer OS and PFS [9]. IDH1 and IDH2 mutations have been shown to correlate with a two- to three-fold increase in survival [30]. However, these mutations occur in only up to 12% of GBMs [31]. Increasingly, IDH mutation and malignant transformation are being linked to numerous alterations at the cellular level [32], e.g., cellular epigenetics, DNA repair pathways, and redox homeostasis [33]. This could provide potential opportunities for targeting these pathways [34] or complementing currently available approved therapeutic protocols [33,35,36]. The epidermal growth factor receptor (EGFR) belongs to the family of epidermal growth factor receptors with tyrosine kinase activity [37]. Dysregulation of the epidermal growth factor signaling pathway can be observed in approximately 80% of high-grade gliomas, resulting either from aberrant expression of EGFR or an EGFR variant (EGFRvIII) [38,39]. Generally, EGFR amplifications are associated with a poorer prognosis [40,41,42].

Of note, various molecular classifications of GBM exist. A frequently used classification is the one described by Verhaak et al. based on gene signatures thereby discriminating four main GBM subtypes: proneural, neural, classical, and mesenchymal [43]. The proneural subtype is more common in younger patients and is associated with a better prognosis [44]. The role of tumor location also appears to play a prognostic role. In particular, the subventricular zone (SVZ) is discussed as an associated site in gliomagenesis and resistance to treatment. Thereby, the SVZ seems to be an independent prognostic factor in glioblastoma [45]. In addition, tumor size (e.g., diameter greater than 5 cm [46,47]), tumors crossing midline, and central tumor locations appear to have a poorer prognosis [46,47,48,49]. Table 1 summarizes various factors associated with the prognosis of glioblastoma.

Besides traditionally well-described correlations, the question of recurrence and progression-free survival remains unresolved. There is still no standard follow-up and no individualization depending on the patient’s risk. Therefore, efforts have been made to combine imaging and tumor characteristics for follow-up and prognostic assessment.

### 1.2. Radio(geno)mics of Glioblastoma

Radiogenomics can be understood as a synthesis of two basic concepts. Possible molecular correlations can be related to a specific radiological phenotype, whereas, on the other hand, it can be shown how a particular genomic variation affects tumor imaging properties [16,58,59]. Radiogenomics defines relationships between image features and molecular markers [16]. Those investigations can be divided into either exploratory or hypothesis-driven types of studies [58]. Exploratory studies compare imaging features with different genomic alterations, whereas hypothesis-driven studies evaluate radiation phenotypes relevant to a particular genetic alteration [60]. Especially in recent years, the application of artificial intelligence (AI) in medicine has increased considerably and is affecting multiple medical specialties [61]. Machine learning (ML) approaches have become a popular and nowadays widespread used technique to extract multiple features by converting for example medical images into mineable high-dimensional feature sets [62], selecting and determining relevant features for further reduction of data complexity, and finally more precise classification and outcome prediction [63,64]. Beyond the scope of manually extracted features, ML allows for an automated extraction of, e.g., first-order statistics, shape-based/textural/wavelet/geodesic features, or tissue probability maps [65]. As high dimensionality may lead to increased model complexity and overfitting issues, reducing dimensionality by feature selection is an essential step that can be performed by methods such as least absolute shrinkage and selection operator (LASSO) or random forests (RF) [66,67]. Based on selected features, various ML approaches can be applied for classification and outcome prediction, e.g., support vector machines (SVM), decision trees (DT), RFs as an extension of DTs, artificial neural networks (ANN), logistic regression (LR), Naïve Bayes (NB), or K-nearest neighbors (KNN). For further details and comparison of those methods, see [68].

### 1.3. Rationale

Supporting interdisciplinary therapy of glioblastoma requires a comprehensive understanding of the patient’s individual risk of tumor progression and recurrence formation to discuss early therapeutic considerations in a comprehensive manner. In that case, noninvasive routine MR clinical studies may provide insight into a likely response to treatment modalities and could aid in decision making for adjusting an individual follow-up regime. However, numerous studies were conducted over a decade that demonstrated numerous correlations of varying degrees. Thus, to provide a comprehensive view of the connection between tumor-related parameters and imaging characteristics, a systematic literature review was conducted to summarize the current status of radiogenomic associations and their prognostic potential that can be considered for optimization of the patient-specific monitoring and treatment strategy.

## 2. Materials and Methods

### 2.1. Eligibility Criteria

The inclusion of registered articles was limited to those written in English, conducted on human subjects, and published in peer-reviewed journals. The period was limited to the years 2011 to 2021. Inclusion criteria for full review were: (1) confirmed diagnosis of GBM (WHO 2016 definition) by a certified neuropathologist; (2) tumor resection without evidence of residual tumor; (3) evaluation of tumor markers or histopathology; (4) evaluation of magnetic resonance imaging sequences. Exclusion criteria were: (1) low-grade glioma and WHO III anaplastic glioma; (2) subtotal tumor resection with residual tumor; (3) animal studies; (4) reviews.

### 2.2. Information Sources and Search Strategy

An extensive web-based search was conducted following the guidelines provided by Preferred Reporting Items for Systematic Reviews and Meta-Analyses (PRISMA). The study was registered in PROSPERO; registration number CRD42022300803. The literature search was conducted using the databases PubMed, Google Scholar, and Cochrane Library. Keywords used were “glioma”, “glioblastoma”, “imaging genomics” and “radiogenomics”. Search terms were combined with two Boolean operators: AND, OR. The search strategy was peer-reviewed by M.B. using the Peer Review of Electronic Search Strategies (PRESS) checklist. The search strategies can be found in the Appendix A (see Table A1).

### 2.3. Selection and Data Collection Process

An extensive web-based search was conducted following the guidelines provided by PRISMA [69] (see Appendix A, Table A2 and Table A3). The PubMed search yielded 121 results, the Google Scholar search 196, and the Cochrane Library 4 results. In total, 321 articles from databases were identified (as of 2 July 2021). As an additional search strategy, references of the selected papers and other reviews were scanned. This led to the identification of additional 30 articles. Titles and abstracts were screened for the inclusion and exclusion criteria by two independent authors (F.C. and D.G.). To increase the consistency among the authors, all authors screened the same publications and discussed the results. If these matched the inclusion criteria, the full-text article was screened for quality using the GRADE criteria and Critical Appraisal Skills Program (CASP) qualitative research checklist. If the quality was judged to be good (meeting three out of four inclusion criteria), the articles were included for the data extraction process. A third author was consulted in case of disagreement between the other two initial authors. Potentially relevant articles were therefore collected in a data table and examined again in consultation with the reviewers. The following information was collected in the consequent data extraction process: (1) author name; (2) publication year; (3) study design; (4) sample size; (5) tumor type; (6) tumor markers; (7) radiological sequences; (8) outcome measure; (9) results. Duplicates were removed. At the end of the selection process, a total of 43 articles was included in this review. A qualitative systematic review was conducted in this setting as numerical data were unavailable in the primarily qualitative studies. Mainly nominal data from the available articles were analyzed.

## 3. Results

**Figure 1:**
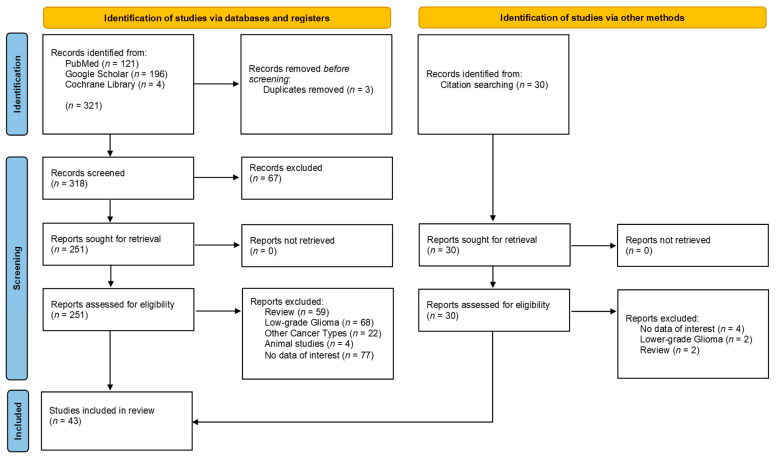
PRISMA flow chart for the systematic review detailing the database searches, the number of records screened, and the studies included.

In total, 321 studies were initially identified. Duplicates (*n* = 3) were removed. Thus, the remaining 318 articles were screened. Reasons for a preliminary exclusion were mainly titles that did not address the research question or were written in a language other than English. A total of 67 articles were excluded. The remaining 251 articles were screened first by abstract, then by full text. Studies were excluded based on the exclusion criteria, e.g., reviews, low-grade gliomas, other tumor types, or animal experiments. Available reviews on this topic were primarily excluded but were searched in a secondary step for valuable literature in the references. Thus, an additional 30 articles of potential relevance were found. These articles went through the same screening process so that a total of 22 more articles were identified here. A total of 43 articles were included in this systematic review. The flow chart of data selection is shown in Figure 1.

Articles were published between 2011 and 2021. The study’s authors, year of publication, molecular parameters, radiological sequences, feature types, and utilization of machine learning are presented in Table 2.

A retrospective study design was predominant (88.37%, *n* = 38). In total, 9176 patients were involved. A heterogeneous, polarizing pattern of cohort sizes, ranging from 6 to 3800 (mean value 382.3, SD 1360.53) was evident. The most evaluated parameters were MGMT (*n* = 22), IDH (*n* = 19), EGFR (*n* = 14), and GBM molecular subgroups (classic, cystic, mesenchymal, and proneural subtype, *n* = 6). Other tumor markers included phosphatase and tensin homolog (PTEN, *n* = 5), platelet-derived growth factor receptor A (PDGFRA, *n* = 5), tumor protein 53 (TP53, *n* = 3), telomerase reverse transcriptase (TERT, *n* = 3), mouse double minute 2 (MDM2, *n* = 3), retinoblastoma protein 1 (RB1, *n* = 3), Ki (Kiel)-antigen Nr. 67 (Ki-67, *n* = 2), mRNA (*n* = 2), signaling pathways (*n* = 2), cyclin dependent kinase 4 (CDK4, *n* = 2), Cyclin Dependent Kinase Inhibitor 2A (CDKN2A, *n* = 2), neurofibromatosis type 1 gene (NF1, *n* = 2), epithelial–mesenchymal transition (EMT) pathway activation (*n* = 1), and mechanistic target of rapamycin (mTOR, *n* = 1).

In addition, the frequency of the radiological MRI sequences used was evaluated. Here, the most frequent sequences were T1 weighted with (*n* = 37) and without contrast agent (*n* = 31), as well as T2-weighted MR sequences (*n* = 33) and fluid-attenuated inversion recovery (FLAIR) sequences (*n* = 26). Other methods included diffusion-weighted imaging (DWI, *n* = 13), incorporating in part parameters such as apparent diffusion coefficient (ADC), fractional anisotropy (FA), radial and axial diffusivity (AD/RD), dynamic susceptibility imaging (DSC, *n* = 12), partially using cerebral blood volume (CBV), cerebral blood flow (CBF), peak height (PH), percentage signal recovery (PSR). Less commonly used modalities were dynamic contrast-enhanced imaging (DCE, *n* = 4), MR spectroscopy (MRS, *n* = 2), arterial spin labeling (ASL, *n* = 1), susceptibility weighted imaging (SWI, *n* = 1), proton density (PD, *n* = 1), spoiled gradient recalled (SPGR) acquisition (*n* = 1) and α-[¹¹C]-methyl-l-tryptophan PET (AMT-PET, *n* = 1).

### 3.1. Studies Assessing the Radiogenomic Prognosis

Five of 43 articles were identified as prospective studies with a total of 321 patients [71,85,88,92,95]. In addition to these articles, other authors also pointed out prognostic implications. [70,73,76,89,105,109]. Evaluations of a retrospective and prospective cohort of patients with newly diagnosed glioblastoma showed that older age, the volume of contrast-enhancing tumor, edema volume, and relatively short distance between the tumor and ventricular system were predictive of shorter survival. In addition, survival was shortened with a relatively high number of voxels of high T1 contrast enhancement intensity, low T2 intensity, high peak height (PH), and low trace (TR) [85]. Heiland et al. demonstrated a strong link between fractional anisotropy (FA) and the epithelial-to-mesenchymal transition (EMT) pathway using network analysis. A high FA correlated with a worse clinical outcome. In contrast, high mean diffusivity (MD) correlated with more prolonged survival [88]. Furthermore, a correlation between a high AMT tumor/cortex uptake ratio on PET and prolonged survival could be observed [92]. Concerning gene and mRNA expression in glioblastoma, Kaplan–Meier curves showed that periostin (POSTN) expression resulted in significantly shorter survival and time to progression [70]. Approaching of the tumor to ADIFFI (analysis of differential involvement)-classified regions in the left temporal lobe significantly prolonged the survival rate [73]. The contrast-enhancing region of the tumor and longest axis length of the tumor were also associated with poor survival [76]. Perfusion imaging showing a high ratio of peri-enhancing tumor area (rCBVperi-tumor) was found in patients with overall survival of less than 14 months. Moreover, the strongest predictors of overall survival were rCBVperi-tumor and age [89].

### 3.2. Studies Assessing the MGMT Methylation Status

Twenty-two out of 43 articles (including 3688 patients) assessed the MGMT methylation status in connection with specific radiological characteristics [72,73,77,78,80,83,84,86,87,91,92,96,98,99,100,101,103,104,105,108,109,111]. In T2/FLAIR images, MGMT promoter-methylated tumors were shown to have a lower hyperintense tumor volume, in contrast to unmethylated tumors [73]. Additionally, elevated transfer constant (K_trans_) levels in MGMT-methylated tumors were observed in MR perfusion [78]. In diffusion imaging, increased minimum ADC values were more likely in MGMT-methylated tumors. A significant reduction in the mean measure of the low ADC value in the two-mixture model histogram seemed to be associated with methylated tumors as well [80]. Metabolic volume and tumor/cortex AMT unidirectional uptake ratios were lower in MGMT-methylated tumors [92]. A significant association between MGMT-methylated tumors and grade of radiographic necrosis was furthermore demonstrated [105].

### 3.3. Studies Assessing the IDH Mutation Status

Nineteen out of 43 articles (including 2875 patients) examined the IDH status in relation to radiologic characteristics [66,71,72,77,83,84,89,92,93,96,98,99,101,103,104,105,108,109,111]. Using MR spectroscopy, increased levels of 2-hydroxyglutarate (2-HG), the product of the neomorphic IDH enzyme activity, were found in IDH1 mutant tumors. The analysis also showed an increase in measured choline and decreased glutathione levels in IDH1 mutant tumors [71]. There was evidence that IDH1 mutant tumors were non-contrast enhancing tumor (nCET) positive. Tumor size and nCET could be used to determine IDH1 mutated tumors with an accuracy of 97.5% [72]. In contrast to a correlation of MGMT methylation and minimum ADC maps [80], Yamashita et al. did not find a significant difference in differentiation between wild-type IDH1 tumor and mutant IDH1 status in their study. However, a significant increase in absolute tumor blood flow, relative tumor blood flow, necrosis area, and percentage of cross-sectional necrosis area inside the enhancing lesion was observed [84]. Moreover, a larger tumor volume in T2-weighted sequences and a higher volume ratio between T2w and T1 sequences with contrast agents were observed in IDH mutant tumors. Additionally, higher mean normalized apparent diffusion coefficient (ADC) values were seen in these tumors [98].

### 3.4. Studies Assessing the EGFR Status

Fourteen out of 43 articles (including 2265 patients) assessed EGFR or epidermal growth factor receptor variant III (EGFRvIII) status in GBM patients [77,81,82,83,86,89,90,92,95,96,97,101,103,106]. A positive EGFRvIII status, an increased relative plasma volume (rVP), and increased relative contrast transfer coefficient parameters were revealed [81]. Furthermore, higher median relative cerebral blood volume (rCBV) and lower percentage signal recovery (PSR) levels were seen in MR perfusion, which was associated with high levels of EGFR amplification. In addition, higher median relative peak height (rPH) levels were associated with EGFRvIII mutation [82]. Bosnyák et al. described lower T1 contrast-enhancing tumor volume, lower T1 contrast/T2 volume, and T1 contrast/PET volume ratios associated with EGFR amplification [92]. Similarly, higher levels of relative contrast enhancement were seen in the presence of EGFR mutations at alanine 289 (EGFRA289D/T/V). Lower T1 and increased T2 signals were also detected in the enhancing tumor region [103].

### 3.5. Studies Assessing the Molecular Subtypes

Overall, seven out of 43 articles involving 775 patients investigated radiogenomic associations related to glioblastoma molecular subtypes [70,75,76,79,85,86,101]. High POSTN and low miR-219 expression could be significantly associated with the mesenchymal GBM subtype [70]. Differences in image morphology were evident with respect to the volume of contrast enhancement, the volume of central necrosis, the combined volume of contrast enhancement and central necrosis, and necrosis concerning the distinction of the mesenchymal and non-mesenchymal subtypes. The volume ratio of T2-weighted hyperintensity to contrast enhancement and central necrosis was significantly lower in the mesenchymal glioblastoma subtype [75]. Similarly, the proneural class showed significantly lower levels of contrast uptake compared to other subtypes. The mesenchymal subtype showed low levels of non-enhanced tumor [76].

### 3.6. Studies Assessing the Tumor Location

**Figure 2:**
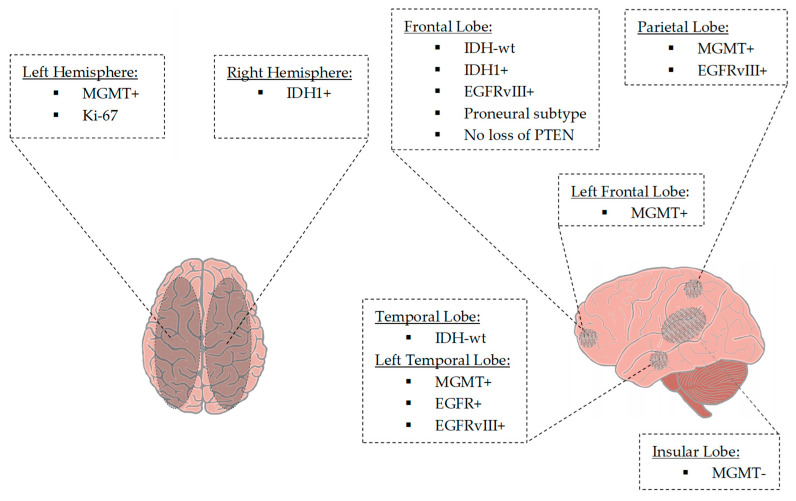
Tumor localization based on tumor-associated parameters. MGMT-methylated tumors and tumors with a lower Ki-67 value were lateralized to the left hemisphere. In contrast, MGMT-unmethylated tumors and tumors with mutation of IDH1 were found to be lateralized to the right hemisphere. IDH1-mutant tumors were located in the frontal lobe, whereas IDH wild-type tumors were located in the frontal and temporal lobes. Tumors with the proneural gene expression subtype and tumors with an absent loss of PTEN frequently occurred in the frontal lobe. MGMT-methylated tumors were located in the (left) frontal and left temporal lobes. In contrast, MGMT-unmethylated tumors were located in the insular lobe. Tumors revealing EGFR amplification and EGFRvIII-mutated tumors involved the frontal, left temporal and parietal regions. Temporal location was associated with IDH wild-type tumors. Abbreviations: EGFR+, EGFR amplification; EGFRvIII+, EGFRvIII mutation; IDH1+, IDH1 mutation; IDH-wt, IDH wildtype; Ki-67, Kiel-antigen Nr.67; MGMT-, MGMT promotor unmethylated; MGMT+, MGMT promotor methylated.

Seven of 43 articles (involving 1557 patients) were able to provide an additional link between radiogenomic markers and tumor location [72,73,77,86,95,96,99]. There was a correlation between nCET positive IDH1 mutant tumors, 79% localized in the frontal lobe [72]. MGMT-methylated tumors having a lower T2/FLAIR volume were localized in the left hemisphere. In contrast, MGMT-unmethylated tumors appeared to be localized in the right hemisphere. T2/FLAIR hyperintense signaling increases frequently occurred in the posterior subventricular zone [73]. Another study showed frequent localization of MGMT methylated tumors in the left temporal lobe. For example, localization in the frontal lobe was seen with younger age, IDH1 mutated tumors, and loss of PTEN. Moreover, in the frontal lobe, localized in the left hemisphere, MGMT-methylated, IDH1-mutated glioblastomas appeared [77]. EGFR amplified, and EGFRvIII-expressing tumors were most common in the left temporal lobe. EGFRvIII-positive tumors occurred more frequently in the frontal and parietal lobe areas than EGFRvIII-negative tumors [95]. Altieri et al. showed a predictive value for left hemisphere occurrence at low Ki-67 levels and IDH1 mutations occurring in the right hemisphere. IDH wild-type glioblastomas appeared to be localized in the temporal lobe. MGMT-methylated tumors occurred mainly in the parietal lobe, whereas unmethylated glioblastomas were localized in the insula [99]. Figure 2 illustrates radiogenomic tumor localizations.

## 4. Discussion

### 4.1. Summary of Findings

Numerous tumor-related molecular markers are used to monitor the treatment of patients with GBM, thereby playing a role in improving diagnostic accuracy, assessing prognosis, and predicting treatment response. The newly established WHO CNS tumor classification 2021 places higher importance on molecular markers than all other previous classifications [11]. In this review, the recent advantages in correlating tumor-associated molecular parameters with imaging phenotypes were highlighted, thereby exhibiting correlative prognostic capabilities of radiogenomics.

MGMT promoter methylation has clinical relevance in the prediction of chemotherapy responsiveness to TMZ [112,113]. MGMT-methylated tumors showed less T2/FLAIR hyperintense volume [73,77] and lower volume contrast enhancement [77] which was associated with the extent of radiographic necrosis [105]. However, Kickingereder et al. described a higher ratio of contrast-enhancing tumor volume (T1CE) to the complete volume (T1w) [86]. Further associations included higher K_trans_ [78], higher relative CBV [86], lower metabolic volume, lower tumor/cortex AMT unidirectional uptake ratios on PET imaging [92], and elevated minimum ADC [80].

IDH1 mutation represents a significant independent factor in predicting longer OS and PFS in patients with GBM [114]. IDH1-mutant tumors revealed elevated levels of 2-HG, a decreased glutathione, and elevated choline in MRS [71]. IDH-mutated tumors revealed higher nADC values, larger volumes in T2w imaging, and a higher ratio between T2w and T1CE [98]. In contrast, IDH wild-type tumors demonstrated larger volumes of contrast enhancement [77], higher blood flow, increased necrotic areas, and a higher percentage of cross-sectional necrosis inside the enhancing lesion [84].

Amplification and alteration of EGFR are frequently observed in GBM, resulting in overexpression of several mutations, including EGFRvIII [41], and was shown to be a predictor of poor prognosis in OS [40,41]. EGFR amplifications were associated with a higher T1CE and T2/FLAIR hyperintense volume [77]. Conversely, Bosnyák et al. found lower T1CE contrast volumes, lower T1 contrast/T2 volume, and T1 contrast/PET volume ratios [92]. EGFR amplification correlated with a higher median rCBV and lower PSR [82], increased rCBF and rCBV [86,89], and a higher Ki-67 labeling index [92]. EGFRvIII expression of tumors was associated with increased rVP [81], relative k_trans_ [81], higher median rPH [82,101], higher rCBV [95,101], FA [101], elevated mean PH within the enhancing tumor [101], and lower ADC signals [95].

Identification of GBM subtypes is of high importance for prognosis as they exhibit different clinical outcomes and molecular features [115]. The mesenchymal subtype was characterized by a high FLAIR signal associated with upregulated levels of POSTN [70], lower ratios of volume of contrast enhancement, and volume of central necrosis [75], lower levels of non-enhanced tumor [76], and lower rCBV [86]. The classical subtype revealed associations concerning T2/FLAIR intensity, enhancing tumor size, and PH signal for edema [79,85] whereas the proneural subtype demonstrated lower levels of contrast enhancement [76] and associations of T1w and T2/FLAIR intensity in the enhancing tumor region [85].

Besides individual biological tumor parameters exhibiting prognostic potential, we identified radiological sequences and tumor locations that were found to be associated with prognosis individually. A high T1CE signal, low T2w intensity, high PH, and low trace (TR) were associated with a shortened survival [85]. FA correlated with the activation of the EMT pathway, thus being associated with a worse clinical outcome [88]. High FLAIR intensity signal was found to correlate with an upregulated POSTN expression level and showed a significantly shortened survival [70]. In contrast, a high MD [88] and a high AMT tumor/cortex ratio on PET corresponded with prolonged survival [92]. A strong predictor for OS was rCBV in the peritumor region [89]. Concerning their location in the CNS, tumors in the left temporal lobe were associated with longer OS independent of treatment and MGMT status [73]. Furthermore, these regions were associated with a more favorable response to radiochemotherapy and increased survival [77].

### 4.2. Limitations

Even though all included studies related to molecular and radiological markers focusing on tumor progression in GBM, one major drawback is the methodological heterogeneity found across the studies. Besides varying the selection of molecular and radiological markers, there are several aspects to consider. Due to the lack of standardization in imaging routines and system-related differences across MRI systems and used sequences, one limitation can be seen in the analysis of the variability and inconsistency in radiological parameters and features. Whereas the used imaging sequences varied across the study, scanner-related variabilities need to be considered when comparing those studies or pooling data in multicenter approaches [116]. In this way, besides other factors, e.g., the magnetic field strength is known to influence image contrast, noise, and tumor-related measures such as lesion contrast [117,118]. Furthermore, significant differences in the methodology could be observed across the included studies, and especially recent ones used AI and ML approaches. In addition, different software with different algorithms was used.

Radiomics and radiogenomics have been associated with AI and ML, especially in recent years. Radio(geno)mic studies can be divided into classical (conventional) and novel approaches with ML [63], representing an essential methodological difference in evaluating and interpreting the study types, as summarized here. The classical approach contrasts to machine learning and deep learning radio(geno)mic pipelines, where images are processed, and features are automatically extracted and related. In the classical approach, the observer’s regions of interest are manually or automatically delineated, and hand-granted features are extracted [63]. Image-derived features are processed, and statistical models like univariate or multivariate analyses are used to calculate mathematical relationships between variables and outcomes [63]. Several of our included studies feature ML and AI algorithms, of which the results cannot be extrapolated for the observer in contrast to the classical radiogenomic approach. Therefore, the results of the individual studies in this review must be interpreted with caution.

### 4.3. Clinical Relevance

In the future, prospective patient cohorts may provide systemic data acquisition concerning radiologic and histopathologic markers. Information on genetic profiles with various biomarkers, structural and functional MR imaging characteristics, and clinical responses related to the type of surgery, could be analyzed. Correlations of these will be analyzed to obtain predictive radiogenomic markers for the prognosis of patients with GBMs with regard to progression, recurrence, and malignancy, enabling them to be used in the future for more personalized and improved treatment planning. This includes a multi-dimensional multi-omic characterization of tumors, i.e., by integrating genomic, proteomic, and radiomic data. To optimize prognosis as well as clinical therapy decisions, there is a need to identify patients at an early stage who are at increased risk for recurrent tumors or low therapeutic success. Relevant radiogenomic characteristics of gliomas can be determined, intratumoral heterogeneity in imaging can be compared with intratumoral genetic heterogeneity, and the differences between primary and secondary glioblastomas as well as markers of tumor angiogenesis can be elaborated. In addition to determining relevant predictive radiogenomic markers for the progression and recurrence of brain-derived tumors, determining prognostically relevant radiogenomic markers, and the formulation of a standard protocol for imaging follow-up concerning the biological characteristics of the tumor, this data collection could allow further analyses. A broad-spectral database thereby created, in conjunction with patients systematically followed up to recurrence, could provide radiogenomic information to identify early tumor recurrence. Such high-risk patients could be monitored more closely in the future, and therapeutic interventions could be considered earlier. Furthermore, a statistically evaluated database of radiogenomic associations can be used for a prognostic scoring system that will be implemented clinically. Similarly, stem cells could be isolated, and different therapy arms (e.g., chemotherapy with temozolomide and radiation) could be analyzed in this regard to achieve improved prognosis and therapy decisions for each patient individually on a broad basis.

### 4.4. Future Directions

Glioblastoma remains in its position as a clinically difficult tumor to treat, yet recent years have demonstrated a steady improvement in imaging modalities, especially in MRI. Attempts to incorporate increasing autonomy into imaging processing programs using AI to create initial prognostic determinations are rising. Radio(geno)mics offers a strong upward trend in recent years and may offer a personalized revolutionary approach in GBM. Likewise, advances in machine learning and deep learning approaches could be observed. New reviews in this field have focused on ML and deep learning approaches [67]. However, in contrast to other reviews, we focused on both classical to machine learning approaches. Our primary objective was to provide a comprehensive summary of radiological parameters and molecular tumor characteristics, thus enabling risk stratification rather than focusing on other aspects such as therapeutic options.

Machine learning algorithms for program-controlled, non-invasive detection of radiogenomic markers in IDH and EGFR in low-grade gliomas and glioblastomas showed success rates of over 80% [101]. Similarly, experiments using anomaly detection analytics detected IDH mutations in glioblastomas using preoperative T1-weighted MR sequences [119]. A neural network-based approach using high-dimensional gene expression data to perform non-linear mapping to imaging traits also showed that imaging features of the tumor exhibited specific transcriptional patterns [120]. Using a hypercolumn-based convolutional network to segment tumor regions from MRI images and extract radiological features such as geometry, shape, and histogram, and finally to fuse them with gene expression profiling data represents another attempt to predict patient survival rates. In this context, the most essential genes identified were, for example, interleukin-1β, KLHL4, ATP1A2, IQGAP2, and TMSL8, which strongly contribute to prognostic analysis [121]. To predict progression-free survival and recurrence, the Cancer Imaging Phenomics Toolkit (CaPTk) software suite was further used to analyze standard clinical multiparametric MRI scans of the brain. Predictive signatures based on various classification schemes were evaluated. These predictors also generated high predictability of the timing and location of recurrence [122]. At the tumor microenvironment level, machine learning-based magnetic resonance radiomodeling was developed to classify immune phenotypes in glioblastoma, which assessed the enrichment levels of four immune subsets. Five immunophenotypes were identified that could also predict patient prognosis [123]. Using a deep learning pipeline to predict MGMT status in glioblastoma patients automatically showed good predictive performance. Using FLAIR images, better status prediction and tumor segmentation could be reached [124]. An essential goal of radiogenomics is to provide prognostic information regarding invasiveness, recurrence, and survival early in the clinical patient course. Many studies can be found in the literature using multicenter databases, for example, The Cancer Genome Atlas (TCGA) and The Cancer Imaging Archive (TCIA). In the future, there is still a lack of clinical, prospective studies that follow up patients with fixed examination times until recurrence, collecting different radiological and tumor biological characteristics at the respective follow-up times, which are finally evaluated by biostatistics using an extensive database.

## 5. Conclusions

The present review provides a broad and informative state-of-the-art picture and illustrates the latest developments in radiogenomic markers with regard to prognosis and their potential monitoring for GBM recurrence. By linking tumor biology parameters with phenotypic characteristics in MR imaging on a patient-specific basis, a significant trend towards a personalized approach is emerging. However, prospective studies analyzing radiogenomic features of glioblastoma are lacking. New information providing answers to prognosis and recurrence early in the course of the disease could provide new clinical implications for individual management and treatment strategies in GBM patients, who are up to now still faced with a poor prognosis and outcome.

## Figures and Tables

**Table 1:** Factors influencing the prognosis in GBM.

Factor	Positive Prognosis	Negative Prognosis	References
EOR	Maximal resection (EOR ≥ 98%)	Subtotal resection (EOR ≤ 78%)	[23,24,25,50,51]
Sex	Female	Male	[17,18,19]
Age	≤40	≥70	[20,21,22]
KPS	≥70	≤60	[21,52]
IDH status	Mutant status	Wildtype status	[30,53,54]
MGMT	Methylated	Unmethylated	[27,28,29]
TERT	No mutation	Mutation	[55,56,57]
EGFR Amplification	Negative	Positive	[40,41,42]
Tumor size	<5 cm (axial diameter)	>5 cm (axial diameter)	[46,47]
Tumor location	Increased distance to center of third ventricle	Crossing midline/ central location	[47,48,49]

Abbreviations: EGFR, epidermal growth factor receptor; EOR, extent of resection; IDH, isocitrate de-hydrogenase; KPS, Karnofsky Performance Sore; MGMT, O6-methylguanine-DNA methyl-transferase; TERT, telomerase reverse transcriptase.

**Table 2:** Literature review table.

Author	Year	Patients (*n*)	Molecular Parameters of Interest	Radiological Sequences of Interest	ML Approach (Classification/Prediction)
Zinn et al. [70]	2011	52	Gene- and micro-RNA expression, molecular subtypes	T1w, T1CE, T2w, FLAIR, PD, SPGR	No
Pope et al. [71]	2012	16	IDH1	MRS	No
Carrillo et al. [72]	2012	202	IDH1, MGMT	T1w, T1CE, T2w	No
Ellingson et al. [73]	2012	258	MGMT	T1w, T1CE, T2w, FLAIR	No
Jamshidi et al. [74]	2013	23	Global mRNA expression and DNA copy number profiles	T1w, T2w	No
Naeini et al. [75]	2013	46	Mesenchymal subtype	T2w, FLAIR	No
Gutman et al. [76]	2013	75	Verhaak’s molecular subtypes	T1w, T1CE, T2w, FLAIR	No
Ellingson et al. [77]	2013	507	IDH1, MGMT, EGFR, PTEN	T1w, T1CE, T2w, FLAIR	No
Ahn et al. [78]	2014	43	MGMT	T1w, T1CE, T2w, DWI (ADC, FA), DCE-MRI (K^trans^, Kep, Ve)	No
Gavaert et al. [79]	2014	55	Molecular subtypes General gene expression	T1w, T1CE, T2w, FLAIR	No
Rundle-Thiele et al. [80]	2015	32	MGMT	DWI (ADC)	No
Arevalo-Perez et al. [81]	2015	82	EGFR amplification/EGFRvIII status	T1CE, DCE-MRI (K^trans^, VP, rVP)	No
Gupta et al. [82]	2015	106	EGFR amplification/EGFRvIII mutation	T2* DSC Perfusion (rCBV, rPH, PSR)	No
Itakura et al. [83]	2015	265	Multiple signaling pathways, MGMT, EGFR, IDH-1, PDGFRA	T1CE	kCC
Yamashita et al. [84]	2016	66	IDH 1, MGMT	T1w, T1CE, T2w, FLAIR, ASL (CBF), DWI (ADC)	No
Macyszyn et al. [85]	2016	134	Verhaak’s molecular subtypes	T1w, T1CE, T2w, FLAIR, DWI (FA, RAD, AD, TR), DSC-MRI (rCBV, PH, PSR)	SVM
Kickingereder et al. [86]	2016	152	Molecular subtypes, MGMT, EGFR, PDGFRA, MDM4, CDK4, PTEN, CDK2A, NF1, Rb1	T1w, T1CE, T2w, FLAIR, DWI (ADC), DSC-MRI (nrCBV, nrCBF), SWI	sGBM, RFC, PLR
Korfiatis et al. [87]	2016	155	MGMT	T1w, T1CE, T2w	SVM, RFC
Heiland et al. [88]	2017	21	EMT pathway activation	T1w, T1CE, T2w, DWI (FA, MD, AD, RD)	No
Liu et al. [89]	2017	41	Ki-67 labeling index, mTOR activation, EGFR amplification, IDH mutation, TP53	T1w, T1CE, T2w, FLAIR, DSC-MRI (CBV, rCBV)	No
Hu et al. [90]	2017	48	EGFR, PDGFRA, PTEN, CDKN2A, RB1, TP53	T1w, T1CE, DSC-MRI (rCBV), DWI (FA, MD, isotropic/anisotropic diffusion)	DTM
Kickingereder et al. [91]	2017	181	MGMT	T1w, T1CE, FLAIR	LASSO
Bosnyák et al. [92]	2018	21	EGFR, MGMT, IDH1	T1w, T1CE, T2w, FLAIR, AMT-PET	No
Liang et al. [93]	2018	102	IDH genotype	T1w, T1CE, T2w, FLAIR	CNN
Beig et al. [94]	2018	115	Hypoxia-associated genes	T1CE, T2w, FLAIR	RFC
Akbari et al. [95]	2018	129	EGFRvIII	T1w, T1CE, T2w FLAIR, DWI (ADC, AD, RD, FA), DSC-MRI (rCBV, PH, PSR)	SVM
Neyra et al. [96]	2018	131	IDH1, MGMT, EGFR, PDGFRA, MDM2, MET, CDK6, TERT, MYCN, NF1, CCND2	T1w, T1CE, T2w, FLAIR	No
Bakas et al. [97]	2018	142	EGFRvIII	T1CE, FLAIR, DWI (FA, RD, AC, TR), DSC-MRI	No
Hong et al. [98]	2018	176	IDH1, MGMT, ATRX	T1w, T1CE, T2w, DWI (ADC), DSC-MRI (CBV)	No
Altieri et al. [99]	2018	178	IDH1, Ki-67, MGMT	T1CE	No
Li et al. [100]	2018	193	MGMT	T1w, T1CE, T2w, FLAIR	RFC
Rathore et al. [101]	2018	261	Verhaak’s molecular subtypes, IDH-1, MGMT, EGFRvIII	T1w, T1CE, T2w, FLAIR, DWI (FA, AD, RD, TR), DSC-MRI (rCBV, PH, PSR), DCE-MRI	SVM
Li et al. [102]	2019	109	PTEN status	T1w, T1CE, T2w	SVM
Binder et al. [103]	2019	260	EGFR, MGMT, IDH	T1w, T1CE, T2w, DWI (ADC, FA, AD, RD), DSC-MRI (rCBV, PH, PSR)	SVM
Le et al. [104]	2020	53	MGMT, IDH1	T1w, T1CE, T2w, FLAIR	XGBoost, kNN, RFC, SVM
Zhang et al. [105]	2020	60	MGMT, IDH, TERT, BRAF	T1w, T1CE, T2w	No
Park et al. [106]	2020	120	EGFR, PDGFRA, MDM2, CDK4, PTEN, p53, CDKI2A, RB1, PIK3CA	T1w, T1CE, T2w, FLAIR, DWI (ADC), DSC-MRI (rCBV, nCBV)	RFC, LRC
Tian et al. [107]	2020	126	TERT	T1w, T1CE, T2w, FLAIR, MRS	LRC
Choi et al. [108]	2020	144	MGMT, IDH	T1w, T1CE, T2w, FLAIR	LASSO-Cox
Beig et al. [109]	2020	203	IDH, MGMT	T1CE, T2w, FLAIR	LASSO-Cox
Zheng et al. [110]	2020	3800	SOCS3, ANGPT1, ANGPT2, FLT1, PECAM1, TEK, TIE1, VEGFA, NRP1, and KDR	DSC-MRI, DCE-MRI	No
Beig et al. [111]	2021	147	MGMT, IDH	T1CE, T2w, FLAIR	LASSO-Cox
Nuechterlein et al. [66]	2021	46	IDH1/2-wildtype	T1w, T1CE, T2w, FLAIR	LASSO. SVM, MLP, XGBoost, RFC, LRC

Abbreviations: AD, axial diffusivity; ADC, apparent diffusion coefficient; AI, artificial intelligence; AMT, α-[11C]-Methyl-l-tryptophan; ANGPT1, angiopoietin-1; ANGPT2, Angiopoietin-2; ATRX, alpha-thalassemia/mental retardation syndrome X-linked; CCND2, cyclin D2; CDK2A, cyclin-dependent kinase inhibitor 2A; CDK4, cyclin-dependent kinase 4; CDK6, cell division protein kinase 6; CNN, convolutional neural network; DCE, dynamic contrast enhanced; DSC, dynamic susceptibility contrast; DTM, decision tree model; DWI, diffusion weighted imaging; EGFR, epidermal growth factor receptor; EGFRvIII, epidermal growth factor receptor variant III; EMT, epithelial–mesenchymal transition; FA, fractional anisotropy; FLAIR, fluid-attenuated inversion recovery; FLT1, Fms-related receptor tyrosine kinase 1; IDH, isocitrate dehydrogenase; kCC, k-means consensus clustering; KDR, kinase insert domain receptor; KEP, rate transfer coefficient; Ki-67, Kiel-antigen Nr. 67; kNN, k-nearest neighbors; K^trans^, transfer constant; LASSO, least absolute shrinkage and selection operator; LASSO-Cox, L1-norm regularized Cox proportional hazard model; LRC, logistic regression classifier; MDM2, murine double minute 2; MET, mesenchymal–epithelial transition factor; MGMT, O6-methylguanine-DNA methyl-transferase; ML, machine learning; MRI, magnetic resonance imaging; MRS, magnetic resonance spectroscopy; mTOR, mammalian target of rapamycin; MYCN, v-myc myelocytomatosis viral-related oncogene, neuroblastoma derived; NF1, neurofibromatosis 1; nrCBF, normalized relative cerebral blood flow; nrCBV, normalized relative cerebral blood volume; NRP1, neuropilin-1; PD, proton density; PDGFRA, platelet-derived growth factor receptor A; PECAM1, platelet endothelial cell adhesion molecule; PLR, penalized logistic regression; PET, positron emission tomography; PSR, percentage signal recovery; PTEN, phosphatase and tensin homologue; RAD, radial diffusivity; RB1, retinoblastoma protein; rCBV, relative blood volume; RFC, random forest classifier; rPH, relative peak height; rVP, relative plasma volume; sGBM, stochastic gradient boosting machine; SOCS3, suppressor of cytokine signaling 3; SPGR, spoiled gradient recalled acquisition; SVM, support vector machine; SWI, susceptibility weighted imaging; T1CE, T1-weighted contrast-enhancement imaging; T1w, T1-weighted imaging; T2w, T2-weighted imaging; TEK, endothelial tyrosine kinase; TERT, telomerase reverse transcriptase; TIE1, tyrosine kinase with immunoglobulin-like and EGF-like domains 1; TP53, tumor protein 53; TR, Trace; Ve, volume fraction of extravascular extracellular space; VEGFA, vascular endothelial growth factor A; VP, plasma volume.

## Appendix A

**Table A1:** PubMed, Google Scholar, and Cochrane Library search strategies.

Database: PubMed	
The search strategy for Title/Abstract terms used a combination of subject headings (MeSH terms) and keywords:	
Search Strategy:	
#1	“GBM”[All fields]
#2	“Glioblastoma”[Mesh]
#3	“Glioma *”[Mesh]
#4	“glioblastoma *”[All fields]
#5	“glioblastoma multiforme”[All Fields]
#6	#1 OR #2 OR #3 OR #4 OR #5
#7	“Imaging Genomic *” [Mesh]
#8	“Radiogenomic *”[All Fields]
#9	#7 OR #8
**#10**	**#6 AND #9**
Database: Google Scholar	
Search Strategy with keywords:	
1	“glioma”
2	“glioblastoma”
3	“imaging genomics”
4	“radiogenomics”
**Full search: “glioma” “glioblastoma” “imaging genomics” “radiogenomics**	
Database: Cochrane Register.	
Search Strategy	
#1	Radiogenomic *
#2	Glioma *
**#3**	**#1 AND #2**

**Table A2:** PRISMA 2020 for Abstracts Checklist.

Section and Topic	Item #	Checklist Item	Reported (Yes/No)
**Title**			
Title	1	Identify the report as a systematic review.	Yes
**Background**			
Objectives	2	Provide an explicit statement of the main objective(s) or question(s) the review addresses.	Yes
**Methods**			
Eligibility criteria	3	Specify the inclusion and exclusion criteria for the review.	Yes
Information sources	4	Specify the information sources (e.g., databases, registers) used to identify studies and the date when each was last searched.	Yes
Risk of bias	5	Specify the methods used to assess risk of bias in the included studies.	No
Synthesis of results	6	Specify the methods used to present and synthesise results.	Yes
**Results**			
Included studies	7	Give the total number of included studies and participants and summarise relevant characteristics of studies.	Yes
Synthesis of results	8	Present results for main outcomes, preferably indicating the number of included studies and participants for each. If meta-analysis was done, report the summary estimate and confidence/credible interval. If comparing groups, indicate the direction of the effect (i.e., which group is favoured).	No
**Discussion**			
Limitations of evidence	9	Provide a brief summary of the limitations of the evidence included in the review (e.g., study risk of bias, inconsistency and imprecision).	Yes
Interpretation	10	Provide a general interpretation of the results and important implications.	Yes
**Other**			
Funding	11	Specify the primary source of funding for the review.	No
Registration	12	Provide the register name and registration number.	No

**Table A3:** PRISMA 2020 Checklist.

Section and Topic	Item #	Checklist Item	Location Where Item Is Reported
**Title**			
Title	1	Identify the report as a systematic review.	Page 1
**Abstract**			
Abstract	2	See the PRISMA 2020 for Abstracts checklist.	Page 1
**Introduction**			
Rationale	3	Describe the rationale for the review in the context of existing knowledge.	Page 4
Objectives	4	Provide an explicit statement of the objective(s) or question(s) the review addresses.	Pages 1–4
**Methods**			
Eligibility criteria	5	Specify the inclusion and exclusion criteria for the review and how studies were grouped for the syntheses.	Page 4
Information sources	6	Specify all databases, registers, websites, organisations, reference lists and other sources searched or consulted to identify studies. Specify the date when each source was last searched or consulted.	Page 4
Search strategy	7	Present the full search strategies for all databases, registers and websites, including any filters and limits used.	Page 4
Selection process	8	Specify the methods used to decide whether a study met the inclusion criteria of the review, including how many reviewers screened each record and each report retrieved, whether they worked independently, and if applicable, details of automation tools used in the process.	Page 4
Data collection process	9	Specify the methods used to collect data from reports, including how many reviewers collected data from each report, whether they worked independently, any processes for obtaining or confirming data from study investigators, and if applicable, details of automation tools used in the process.	Page 4
Data items	10a	List and define all outcomes for which data were sought. Specify whether all results that were compatible with each outcome domain in each study were sought (e.g., for all measures, time points, analyses), and if not, the methods used to decide which results to collect.	Page 4–5
	10b	List and define all other variables for which data were sought (e.g., participant and intervention characteristics, funding sources). Describe any assumptions made about any missing or unclear information.	Page 4–5
Study risk of bias assessment	11	Specify the methods used to assess risk of bias in the included studies, including details of the tool(s) used, how many reviewers assessed each study and whether they worked independently, and if applicable, details of automation tools used in the process.	n.a.
Effect measures	12	Specify for each outcome the effect measure(s) (e.g., risk ratio, mean difference) used in the synthesis or presentation of results.	n.a.
Synthesis methods	13a	Describe the processes used to decide which studies were eligible for each synthesis (e.g., tabulating the study intervention characteristics and comparing against the planned groups for each synthesis (item #5)).	Page 5
	13b	Describe any methods required to prepare the data for presentation or synthesis, such as handling of missing summary statistics, or data conversions.	n.a.
	13c	Describe any methods used to tabulate or visually display results of individual studies and syntheses.	n.a.
	13d	Describe any methods used to synthesize results and provide a rationale for the choice(s). If meta-analysis was performed, describe the model(s), method(s) to identify the presence and extent of statistical heterogeneity, and software package(s) used.	n.a.
	13e	Describe any methods used to explore possible causes of heterogeneity among study results (e.g., subgroup analysis, meta-regression).	n.a.
	13f	Describe any sensitivity analyses conducted to assess robustness of the synthesized results.	n.a.
Reporting bias assessment	14	Describe any methods used to assess risk of bias due to missing results in a synthesis (arising from reporting biases).	n.a.
Certainty assessment	15	Describe any methods used to assess certainty (or confidence) in the body of evidence for an outcome.	n.a.
**Results**			
Study selection	16a	Describe the results of the search and selection process, from the number of records identified in the search to the number of studies included in the review, ideally using a flow diagram.	Page 5
	16b	Cite studies that might appear to meet the inclusion criteria, but which were excluded, and explain why they were excluded.	n.a.
Study characteristics	17	Cite each included study and present its characteristics.	Pages 6, 9
Risk of bias in studies	18	Present assessments of risk of bias for each included study.	n.a.
Results of individual studies	19	For all outcomes, present, for each study: (a) summary statistics for each group (where appropriate) and (b) an effect estimate and its precision (e.g., confidence/credible interval), ideally using structured tables or plots.	Pages 9–12
Results of syntheses	20a	For each synthesis, briefly summarise the characteristics and risk of bias among contributing studies.	n.a.
	20b	Present results of all statistical syntheses conducted. If meta-analysis was done, present for each the summary estimate and its precision (e.g., confidence/credible interval) and measures of statistical heterogeneity. If comparing groups, describe the direction of the effect.	n.a.
	20c	Present results of all investigations of possible causes of heterogeneity among study results.	n.a.
	20d	Present results of all sensitivity analyses conducted to assess the robustness of the synthesized results.	n.a.
Reporting biases	21	Present assessments of risk of bias due to missing results (arising from reporting biases) for each synthesis assessed.	n.a.
Certainty of evidence	22	Present assessments of certainty (or confidence) in the body of evidence for each outcome assessed.	n.a.
**Discussion**			
Discussion	23a	Provide a general interpretation of the results in the context of other evidence.	Page 12
	23b	Discuss any limitations of the evidence included in the review.	Page 13
	23c	Discuss any limitations of the review processes used.	Page 13
	23d	Discuss implications of the results for practice, policy, and future research.	Pages 13–14
**Other Information**			
Registration and protocol	24a	Provide registration information for the review, including register name and registration number, or state that the review was not registered.	Page 4
	24b	Indicate where the review protocol can be accessed, or state that a protocol was not prepared.	n.a.
	24c	Describe and explain any amendments to information provided at registration or in the protocol.	n.a.
Support	25	Describe sources of financial or non-financial support for the review, and the role of the funders or sponsors in the review.	Page 16
Competing interests	26	Declare any competing interests of review authors.	Page 16
Availability of data, code and other materials	27	Report which of the following are publicly available and where they can be found: template data collection forms; data extracted from included studies; data used for all analyses; analytic code; any other materials used in the review.	n.a.
